# 2134. Impact of ADC ß-lactamase amino acid variation on cefiderocol (FDC) susceptibility in carbapenem-resistant *Acinetobacter baumannii* (CR*Ab*) strains

**DOI:** 10.1093/ofid/ofad500.1757

**Published:** 2023-11-27

**Authors:** Alina Iovleva, Christi McElheny, Ellen G Kline, Robert A Bonomo, Steven Smoke, Marco Falcone, Ryan K Shields, Yohei Doi

**Affiliations:** University of PIttsburgh, Pittsburgh, Pennsylvania; University of Pittsburgh, Pittsburgh, Pennsylvania; University of Pittsburgh, Pittsburgh, Pennsylvania; Louis Stokes Cleveland Department of Veterans Affairs Medical Center, Cleveland, Cleveland, Ohio; Cooperman Barnabas Medical Center, Livingston, New Jersey; Infectious Diseases Unit/University of Pisa, Pisa, Toscana, Italy; University of Pittsburgh, Pittsburgh, Pennsylvania; University of Pittsburgh, Pittsburgh, Pennsylvania

## Abstract

**Background:**

Infections due to CR*Ab* cause high mortality in hospitalized patients. Treatment options are limited, but now include FDC, a novel siderophore cephalosporin. Following clinical implementation, cases of treatment-emergent resistance have been reported. Proposed resistance mechanisms include alterations in iron transporters and amino acid substitutions in *Acinetobacter*-derived AmpC β-lactamase (ADC). Our objective was to define the relative contribution of ADC amino acid substitution to reduced FDC susceptibility against CR*Ab* clinical strains.

**Methods:**

Variant ADCs were identified from whole genome sequencing analysis of clinical CR*Ab* isolates from the US and Italy. We compared common variants (ADC-30, - 33, -56, -73) to variants from FDC-resistant strains that had not been exposed to FDC (ADC-224, -225, -227), and variants from FDC-resistant strains collected following FDC treatment (ADC-30 with V298E and S286R substitutions, ADC-73 with R148Q substitution). *bla*_ADC-30_ was used as a reference. The *bla_ADC_* genes with an upstream ribosomal binding site from pET24 were subcloned into pBCSK (-), transformed into *E. coli* Top10 cells, and selected on plates containing 100 µg/ml ampicillin and 30 µg/ml chloramphenicol. The constructs’ sequences were verified by Sanger sequencing. Minimum inhibitory concentrations (MICs) of FDC were determined by broth microdilution in iron-depleted media at least in duplicate.

**Results:**

Among studied ADC sequences, substitutions that were located in the Ω- or R2-loop regions, which accommodate the binding of cephalosporins, resulted in a 2- to 8-fold change in FDC MIC (Figure 1). When both regions were affected, there was an 8- to 16-fold increase in MIC. No significant MIC changes occurred among the studied mutations outside of these regions (Table 1). R2-loop substitutions were observed in FDC-exposed resistant strains, whereas Ω-loop mutations were observed in resistant strains that had not been exposed to FDC.

Figure 1.

**Figure:**
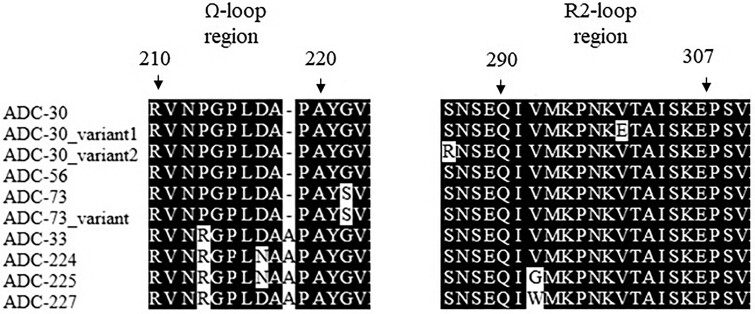

Alignment of amino acid sequences in the ADC Ω- and R2- loop regions among studied isolates.

**Figure:**
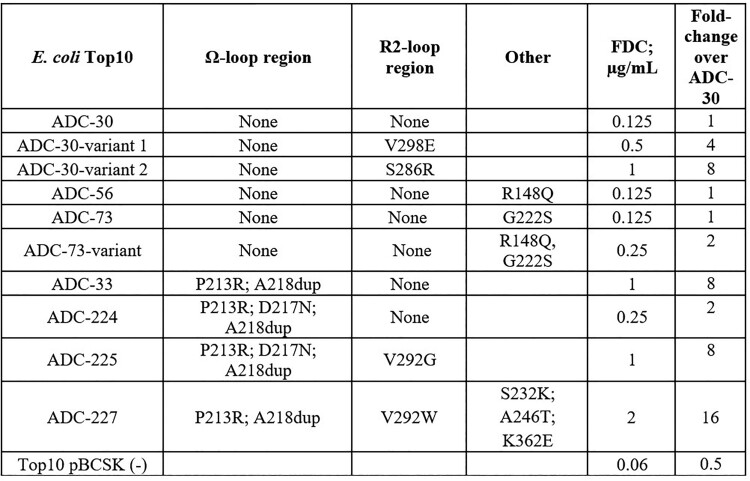

**Conclusion:**

We identified amino acid substitutions in Ω- and R2-loop regions of ADC that contribute to increased FDC MIC. Given the broad diversity of currently circulating ADC variants, it is crucial to study the impact of sequence variation on the activity of novel agents.

**Disclosures:**

**Robert A. bonomo, MD**, Entasis, Merck, VenatoRx, Wockhardt: Grant/Research Support **Marco Falcone, MD, PhD**, Gilead: Board Member|Gilead: Honoraria|Menarini: Board Member|Menarini: Grant/Research Support|Menarini: Honoraria|MSD: Board Member|MSD: Grant/Research Support|MSD: Honoraria|Nordic Pharma: Honoraria|Pfizer: Board Member|Pfizer: Honoraria|Shionogi: Honoraria **Ryan K. Shields, PharmD, MS**, Allergan: Advisor/Consultant|Cidara: Advisor/Consultant|Entasis: Advisor/Consultant|GSK: Advisor/Consultant|Melinta: Advisor/Consultant|Melinta: Grant/Research Support|Menarini: Advisor/Consultant|Merck: Advisor/Consultant|Merck: Grant/Research Support|Pfizer: Advisor/Consultant|Roche: Grant/Research Support|Shionogi: Advisor/Consultant|Shionogi: Grant/Research Support|Utility: Advisor/Consultant|Venatorx: Advisor/Consultant|Venatorx: Grant/Research Support **Yohei Doi, MD, PHD**, bioMerieux: Advisor/Consultant|FujiFilm: Advisor/Consultant|Gilead: Advisor/Consultant|Gilead: Honoraria|GSK: Advisor/Consultant|Meiji Seika Pharma: Advisor/Consultant|Moderna: Advisor/Consultant|Moderna: Honoraria|MSD: Advisor/Consultant|MSD: Honoraria|Shionogi: Advisor/Consultant|Shionogi: Grant/Research Support|Shionogi: Honoraria

